# Error Analysis and Experimental Study of a Bi-Planar Parallel Mechanism in a Pedicle Screw Robot System

**DOI:** 10.3390/s16122022

**Published:** 2016-11-30

**Authors:** Qingjuan Duan, Zhijiang Du, Hongjian Yu, Yongfeng Wang, Wei Dong

**Affiliations:** 1 School of Mechano-Electronic Engineering, Xidian University, Xi’an 710071, China; qjduan@xidian.edu.cn; 2 State Key Laboratory of Robotics and System, Harbin Institute of Technology, Harbin 150001, China; yuhongjian99@126.com (H.Y.); wang_yf16@163.com (Y.W.); dongwei@hit.edu.cn (W.D.)

**Keywords:** robot-assistant spine surgery, bi-planar 5R mechanism, error analysis, dimension error, joint angle error, non-parallelism of the rotation axis

## Abstract

Due to the urgent need for high precision surgical equipment for minimally invasive spinal surgery, a novel robot-assistant system was developed for the accurate placement of pedicle screws in lumbar spinal surgeries. The structure of the robot was based on a macro-micro mechanism, which includes a serial mechanism (macro part) and a bi-planar 5R parallel mechanism (micro part). The macro part was used to achieve a large workspace, while the micro part was used to obtain high stiffness and accuracy. Based on the transfer function of dimension errors, the factors affecting the accuracy of the end effectors were analyzed. Then the manufacturing errors and joint angle error on the position-stance of the end effectors were investigated. Eventually, the mechanism of the strain energy produced by the deformation of linkage via forced assembly and displacements of the output point were calculated. The amount of the transfer errors was quantitatively analyzed by the simulation. Experimental tests show that the error of the bi-planar 5R mechanism can be controlled no more than 1 mm for translation and 1° for rotation, which satisfies the required absolute position accuracy of the robot.

## 1. Introduction

**Figure 1:**
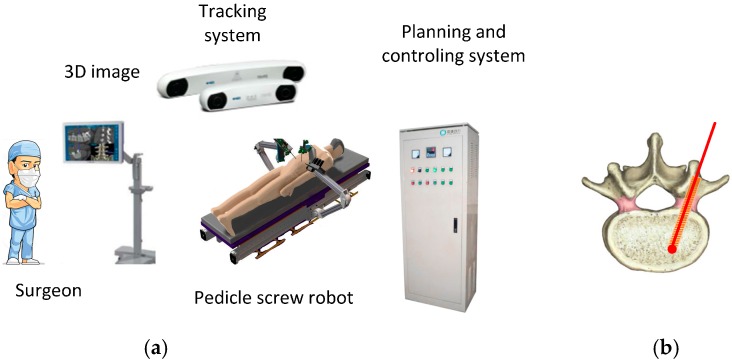
Sketch of the pedicle screw robot system. (**a**) The structure of the pedicle screw robot system; (**b**) Surgery enlarged view.

In the placement of pedicle screws, the screw is implanted in a narrow path as shown in Figure 1. If the screw path is inaccurate to a certain degree, skew or vertebra breakthrough may occur, causing serious vascular and neurological damage to patients, e.g., spinal damage to the medula can lead to paraplegia. Surgeons inevitably experience fatigue during traditional tedious surgical procedures, and this fatigue and hand tremors can give rise to a high accident rate, even for the experienced surgeons [1,2,3,4].

To deal with these issues, computer-assistant surgery devices, a huge shift in clinical operations, have been developed in recent years. RoboDoc is an integrated Surgical Robot Systems approved by the Food and Drug Administration (FDA) [5,6]. Renaissance [7], a bone-mounted 6-degrees of freedom (dof) miniature robot, launched by Mazor Robotics Ltd, is the only spine surgical robot applied in clinical operations. A new type of pedicle screw robot system [8], developed by Shenzhen Institutes of Advanced Technology, contains a 5-degrees of freedom (dof) mechanical arm and a bone screw implanting device. A regional control algorithm was applied to guarantee the safety of the surgery process.

A new type of macro-micro [4] pedicle screw mechanism was proposed to help surgeons precisely handle the insertion of the screws. The parallel micro mechanism plays a key role in accurately manipulating the position and orientation of the movement.

Accuracy is one of the most important performance indices in pedicle screw surgical applications. The accuracy of the parallel mechanisms has been investigated in [9,10,11,12]. Like in traditional machine tools, there are several major factors that influence the precision of parallel mechanisms; the main errors are as follows [13]: (1) Original manufacturing errors; (2) assembly errors; (3) errors resulting from distortion caused by force and heat; (4) control system errors and actuator errors and (5) other stochastic errors [14]. If these errors were to be transferred and accumulated, the position-stance of end-effector may be out of control. Therefore, the robots cannot be controlled precisely and operational errors would increase greatly. In order to minimize the errors of bi-planar parallel mechanisms, the manufacture and assembly precision must be improved. 

In order to meet the design requirements, the essential errors produced and their influence on the end effecter must be analyzed. In this paper, firstly, the dimension error transfer function was analyzed, then the transmission coefficient of each link was proposed. Secondly, the manufacturing errors of each member and its effect on the position-stance of the end effectors were analyzed. Thirdly, the influence of joint clearance for the position error was discussed. At last, the additional internal force of mechanism and deformation of link were calculated by using the the strain energy method.

The remainder of this paper is organized as follows: Section 2 presents the structure of the pedicle screw robot system. In Section 3, the error analysis of the bi-planar mechanism was given in details. Positioning and orientation accuracy experiments were carried out in Section 4. Conclusions are presented in Section 5.

## 2. Structure and Working Principle of the Pedicle Screw Robot System

### 2.1. Structure Overview

The pedicle screw robot system consists of the 3D image information system, the tracking system, the robot, the C-arm X-ray equipment, and the planning and control system as shown in Figure 1. Prior to the operation, the robot and the vertebra must be calibrated with respect to their tracking markers. The tracking system was equipped with a stereo camera. Based on the 3D image information that the tracking system collected, the navigation system was used to calculate the optimal route of drilling, and guide the bone screw implantation. The system can accomplish complex tasks according to the feedback of sensors.

**Figure 2:**
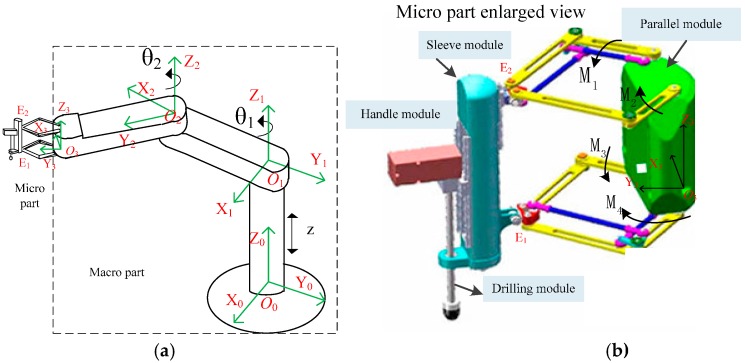
Schematic of the macro-micro mechanism and the parallel robot. (**a**) Structure of the macro-micro mechanism; (**b**) Micro part enlarged view.

The structure of the pedicle screw robot is based on the macro-micro mechanism [4,8]. An attached bi-planar parallel mechanism is shown in Figure 2a. The macro part, shown in the dotted box in Figure 2a, is composed of a 3-dof serial mechanical arm to achieve a large workspace, which is designed according to the demands of the operating room environment. The two rotation joints are *θ*_1_ and *θ*_2_. The translation joint is *z* along the *z*_0_ axis. The micro part is a bi-planar parallel mechanism attached on the end of the macro part, which is used to ensure high stiffness and accuracy. Two independent mechanisms are used to guarantee the safety and the stability for the clinical operation. 

A detailed view of the bi-planar parallel robot is shown in Figure 2b. It includes the bi-planar parallel module, the sleeve module, and the handle module. E1 and E2 are the connecting points on the sleeve model. The lower planar is connected to E1 on the sleeve model by a universal joint. The upper planar is connected to the sleeve model though a universal joint and a cylindrical joint. E2 will move up and down along the cylindrical joint when the sleeve model is tilted. The micro part has 4-degrees of freedom (dofs), i.e., 2-dofs for positioning in X_3_O_3_Y_3_ plane, two rotational dofs about the X_3_ and Y_3_ axis, and with extra actuators which has one dof for rotating and one dof for feeding. The driven joints (M_1_ to M_4_) are shown in Figure 2b. A detailed mechanism description can be found in [15]. The double parallelogram mechanism formed by the joint bearings and the links is designed to increase the stiffness of the parallel mechanism as shown in Figure 2b. 

### 2.2. Working Strategy

In the operation process, the macro part of the surgical robot can realize the preliminary positioning task in the work zone, while the bi-planar mechanism can realize the fine positioning and operation tasks afterwards. Operation steps of the entire system are as follows: firstly, before the robot tip contacts the vertebra, the macro part, 3-dofs serial robot, moves into the work zone; secondly, the micro part, the bi-planar robot, begins to work when the pose error of the serial robot is within a certain tolerance. The drilling process can be carried out either manually or automatically. Different modules can be connected through a quick change device to guarantee the stability and quickness of the operation. The required absolute positioning accuracy of the robot combined with the navigation system for the placement of pedicle screws is millimeter-scale [16]. 

In addition, variations of two grading scales are currently used to describe pedicle screw placement. One is the Gertzbein classification [17,18], in which cortical breaches are described by the extent of extracortical screw violation. In this system, Grade 0 screws are those that are fully contained within a pedicle with no evidence of cortical breach; Grade 1 screws breached 2 mm or less; Grade 2 screws breached 2 mm to 4 mm; Grade 3 screws breached more than 4 mm. Grade 0 and Grade 1 are accurate placements. the other is the Heary classification [19]: Grade I, screw entirely contained within pedicle; Grade II, violates lateral pedicle but screw tip entirely contained within the vertebral body (VB); Grade III, tip penetrates anterior or lateral VB; Grade IV, breaches medial or inferior pedicle; and Grade V, violates pedicle or VB and endangers spinal cord, nerve root, or great vessels and requires immediate revisions.

The technical specifications are established according to: (1) The accuracy of the surgical requirement [17,18,19,20,21] and the clinical effect [22]; (2) 1 mm and 1 degree of error for parallel mechanism are the best accuracy from the current manufacturing and processing technology in our cooperative enterprise. Therefore, taking into account the above factors and [23], the specifications of the bi-planar 5R parallel mechanism of surgical robot are 0 ± 1 mm in position and 0 ± 1° in orientation, separately. The required working space is to cover two lumbar spines so the working space radium of lower plane is set at 50 mm, and the angle of implanting screw is less than 30° [24].

## 3. The Error Analysis

The bi-planar parallel robot is considered to be more advantageous because of its simple structure and high rigidity. However, dimensional errors inevitably occur during manufacture and assembly, which affect the accuracy of the end-effecter motion. Based on the error transfer function and constraints, the influence of dimensional errors, the joint angles, and non-parallelism of the rotation axis deformation compatibility conditions of planar 5R parallel robot were investigated. The additional internal force of mechanism and deformation of link were calculated by the matrix force method, and then the strain energy fluctuations were analyzed.

The pose of sleeve model (E1, E2) of the bi-planar parallel robot, as shown in Figure 2b, is determined by the position of the upper and lower planar 5R mechanism. Hence the planar 5R mechanism is chosen as the research target. Since the planar 5R mechanism is composed of four links through the revolute joints, the errors depend mainly on the dimensional precision, the joint clearances, and the non-parallelism of the rotation axis. 

In order to completely characterize the errors of the planar 5R mechanism, an error transfer function was firstly introduced. Then, elastic deformation energy equations caused by the non-parallelism of the rotation axis were obtained.

### 3.1. Error Transfer Function

The positioning error of the planar 5R mechanism is related to the kinematics of the mechanism [25]. Under the ideal conditions, the position of the output point *P* is given below:

(1) $$P_{k}^{0}=P_{k}^{0}\left(x_{e}\right)=P_{k}^{0}\left(\varphi_{i},r_{j}\right)=P_{k}^{0}\left(\varphi_{1},\varphi_{2},\cdots \varphi_{n},r_{1},r_{2},\cdots r_{m}\right)$$

where $P_{k}^{0}$ is the position of the *k*th driven member. $x_{e}$ is the abbreviation of $\varphi_{i}$, $r_{j}$ is defined as the value of the *i*th generalized coordinates, and $r_{j}$ is defined as the value of the *j*th size parameter. *n* is the drive number, and m is the number of the member in the mechanism.

The actual position of the output point is given by:

(2) $$P_{k}=P_{k}\left(x_{e}+\Delta x_{e}\right)$$

where $\Delta x_{e}$ is the error between the actual and ideal position. 

The Equation (2) is expanded by Taylor series:

(3) $$P_{k}=P_{k}\left(x_{e}\right)+\sum_{r=1}^{n}\frac{\partial P_{k}}{\partial x_{e}}\Delta x_{e}+\frac{1}{2!}\sum_{p=1}^{N}\sum_{q=1}^{N}\frac{\partial^{2}P_{k}}{\partial x_{p}\partial x_{q}}\Delta x_{p}\Delta x_{q}$$

where $\frac{\partial P_{k}}{\partial x_{e}}$ is the error transfer function. Ignoring higher-order item, the Equation (2) is simplified as:

(4) $$P_{k}=P_{k}\left(x_{e}\right)+\sum_{r=1}^{n}\frac{\partial P_{k}}{\partial x_{e}}\Delta x_{e}$$

Total position error is:

(5) $$\Delta P_{k}=P_{k}-P_{k}^{0}=P_{k}\left(x_{e}\right)-P_{k}^{0}+\sum_{r=1}^{n}\frac{\partial P_{k}}{\partial x_{e}}\Delta x_{e}$$

The design error is approximated to zero, and the final form of the position error is:

(6) $$\Delta P_{k}=\sum_{r=1}^{n}\frac{\partial P_{k}}{\partial x_{e}}\Delta x_{e}=\sum_{i=1}^{n}\frac{\partial P_{k}}{\partial \varphi_{i}}\Delta \varphi_{i}+\sum_{j=1}^{m}\frac{\partial P_{k}}{\partial r_{j}}\Delta r_{j}=J\Delta \varphi +\sum_{j=1}^{m}\frac{\partial P_{k}}{\partial r_{j}}\Delta r_{j}$$

where, the error function *J* can also be seen as the jacobian if we are assuming *ϕ* are joint values. It is commonly know that the largest delta *P* happens along the longest principal axis of the manipulability hype-ellipsoid of matrix *J* [26]. 

### 3.2. Effect of Non-Parallelism of the Rotation Axis

The planar 5R mechanism is a closed kinematic chain and overconstrained mechanism [27]. A special geometric constraint, joint axes parallel to each other, is necessary to achieve the end-effecter motion. Closed kinematic chains are considered to be more advantageous in rigidity, power-output, and accuracy than open kinematic chains. However, it must be stressed that closed kinematic chains are so sensitive to machining errors that a tenth of a millimeter of error might result in jamming and immobility [28]. Moreover, overconstrained mechanisms have the essential drawback of sensitivity with respect to the geometrical conditions. Therefore, constraint errors can produce a series of bad effects on the mechanisms. Based on overconstraints analysis, the deformation compatibility conditions of planar 5R parallel robot, geometric constraint errors of joint axes were taken into consideration in the investigation.

Three overconstraints of a planar 5R mechanism are *θ_x_*, *θ_y_*, *S_z_* [29] (*S* is the translation along the coordinate axis, *θ* is the rotation around the coordinate axis; subscripts indicate the direction of movement or the rotating axis), respectively. The above constraint is the result of the interaction of the five rotation pairs of parallel axes. When the parallel constraint conditions are not satisfied, motion error is generated between the connecting rod and the pair element. In addition, elastic deformation is generated to compensate the closed chain mechanism. There will be induced force along the *z* axis and moments rotating the *x* and *y* axis. The additional force and moments cannot make the mechanism smoothly or even stuck.

**Figure 3:**
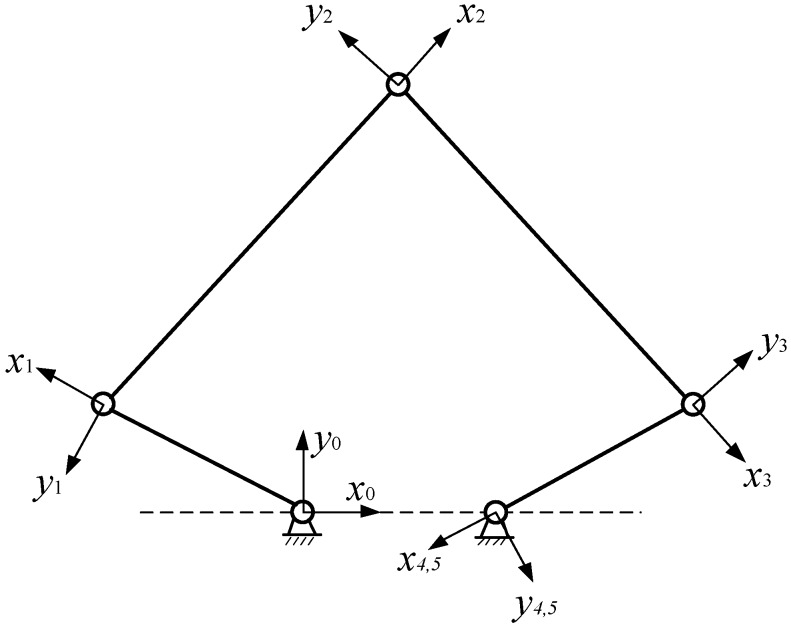
5R coordinate system of the 5R mechanism.

In order to analyze the deformation coordination caused by constraint errors, the local coordinate system was established at the end of each rod in the error-free state, as shown in Figure 3.

The local coordinate system of each link is established, positive direction of axis *x* is defined along the direction of the link, and the rotation axis direction is *z* axis. The coordinate system *x*_0_*y*_0_ is a fixed coordinate system. The right side of the mechanism is disconnected from the right side of the frame under any pose, and the coordinate system of this point is coincident with the fourth local coordinate system in the fixed coordinate system. Based on the coordinate transformation method, the expression is given as:

(7) $$T_{5}^{0}=T_{4}^{0}$$

**Figure 4:**
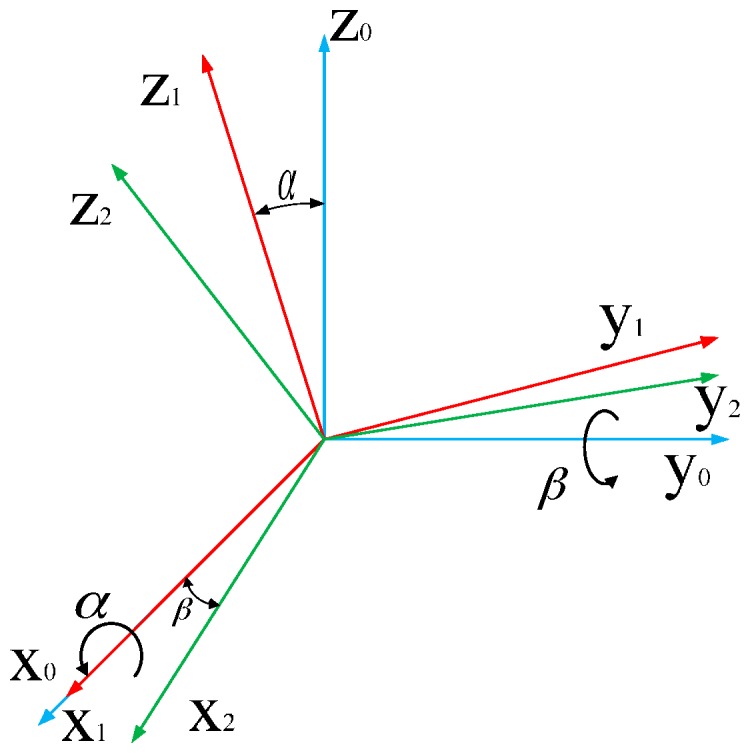
Sketch of $\alpha_{i}$ and $\beta_{i}$.

Under this assumption, the whole mechanism is divided into a 4R open chain mechanism and a connection link assembled to the frame. Because the manufacturing process cannot be strictly parallel to the design axis, local coordinate of the end pose of the 4R mechanism is

(8) $$T_{4a}^{0}=\prod_{i=1}^{4}T_{i}^{i-1}\Delta_{i}=T_{4}^{0}\Delta$$

where $\Delta_{i}$ is differential motion caused by the parallel degree error of the rotation axis of the *i*th link, and Δ is the differential movement of the end link. They are expressed as:

$$\Delta_{i}=\left\{\begin{array}{cccc} 1 & 0 & \beta_{i} & 0\\ 0 & 1 & -\alpha_{i} & 0\\ -\beta_{i} & \alpha_{i} & 1 & 0\\ 0 & 0 & 0 & 1 \end{array}\right\}\text{ and }\Delta =\left\{\begin{array}{cccc} 1 & -\delta_{x} & \delta_{y} & P_{x}\\ \delta_{z} & 1 & -\delta_{x} & P_{y}\\ -\delta_{y} & \delta_{x} & 1 & P_{z}\\ 0 & 0 & 0 & 1 \end{array}\right\}$$

in which $\alpha_{i}$ is the axis phase angle about *x*_0_; $\beta_{i}$ is the axis intersection angle about *y*_0_, as shown in Figure 4.

When the drive pair axis 5 is non-parallel to the rotation axis *x*_0_, the local coordinate of the disconnected point can be expressed as:

(9) $$T_{5a}^{0}=T_{5}^{0}\Delta_{5}$$

When the planar 5R mechanism is closed, according to Equations (7)–(9), there is a transformation matrix *T*e, which makes the final coordinate system coincide with the revolute joint of the connecting frame. *T*e can be expressed as:

(10) $$T_{e}={\left(T_{4a}^{0}\right)}^{-1}T_{5a}^{0}={\left(T_{4}^{0}\Delta \right)}^{-1}(T_{5}^{0}\Delta_{5})=\left(\begin{array}{cccc} 1 & -\gamma & \beta & d_{x}\\ \gamma & 1 & -\alpha & d_{y}\\ -\beta & \alpha & 1 & d_{z}\\ 0 & 0 & 0 & 1 \end{array}\right)$$

where *α*, *β*, *γ*, *d_x_*, *d_y_*, *d_z_* indicate the rotation angles and displacements of each coordinate axes of the 4th link end coordinate to the 4th link coordinate. It is the compatible displacement when the mechanism is closed.

In order to facilitate the calculation, the parameters are transferred to the coordinate system. The transformation formulas can be expressed as:

(11) $$\left\{\begin{array}{c} \alpha_{0}\\ \beta_{0}\\ \gamma_{0} \end{array}\right\}=R_{4a}^{0}\left\{\begin{array}{c} \alpha \\ \beta \\ \gamma \end{array}\right\}\left\{\begin{array}{c} x_{0}\\ y_{0}\\ z_{0} \end{array}\right\}=R_{4a}^{0}\left\{\begin{array}{c} d_{x}\\ d_{y}\\ d_{z} \end{array}\right\}$$

where $R_{4a}^{0}$ is the rotation matrix part of the matrix $T_{4a}^{0}$.

Because the mechanism has the degrees of freedom along the *xoy* plane and around the *z* axis, *x*_0_, *y*_0_ and *γ*_0_ can be compensated by the mechanism motion. The other movement errors are restricted by the constraints of the mechanism, which need to be compensated by the forced deformation of the component. Therefore, *α*_0_, *β*_0_ and *z*_0_ are the deformation coordinates caused by constraint errors.

The parallelism errors in the planar 5R mechanism lead to forced deformation of the assembly component, including the movement along the *z* direction, rotation around the *x* axis and the *y* axis, which correspond to the forced generalized force *F*_Z_ along the z direction, moments *M_x_*, *M_y_* around the *x* axis, and *y* axis, respectively. These forces disturbed the motion of the mechanism. 

If the frame and joint are regarded as a rigid body, the mechanism is equivalent to a planar grid structure. The links are regarded as cantilever beams, and the bending and torsion deformation are combined. Then the 4R open-end mechanism of the end of deformation coordination matrix equation can be written as follows:

(12) $$\left\{\begin{array}{ccc} \delta_{11} & \delta_{12} & \delta_{13}\\ \delta_{21} & \delta_{22} & \delta_{23}\\ \delta_{31} & \delta_{32} & \delta_{33} \end{array}\right\}\left\{\begin{array}{c} F_{z}\\ M_{x}\\ M_{y} \end{array}\right\}=\left\{\begin{array}{c} z_{0}\\ \alpha_{0}\\ \beta_{0} \end{array}\right\}$$

where $\delta_{ij}$ is the flexibility coefficient. The matrix is the flexibility matrix, and the inverse matrix of the flexibility matrix is the stiffness matrix.

Assuming any section of the bar is exactly the same, and is a straight link. According to the material mechanics [30], the flexibility matrix of the link *i*th is expressed as:

(13) $$C_{ii}=\left[\begin{array}{cccccc} \frac{l_{i}}{E_{i}S_{i}} & 0 & 0 & 0 & 0 & 0\\ 0 & \frac{{l_{i}}^{3}}{3E_{i}I_{iz}} & 0 & 0 & 0 & \frac{{l_{i}}^{2}}{2E_{i}I_{iz}}\\ 0 & 0 & \frac{{l_{i}}^{3}}{3E_{i}I_{iy}} & 0 & \frac{{l_{i}}^{2}}{2E_{i}I_{iy}} & 0\\ 0 & 0 & 0 & \frac{l_{i}}{G_{i}J_{i}} & 0 & 0\\ 0 & 0 & -\frac{{l_{i}}^{2}}{2E_{i}I_{iy}} & 0 & \frac{l_{i}}{E_{i}I_{iy}} & 0\\ 0 & \frac{{l_{i}}^{2}}{2E_{i}I_{iz}} & 0 & 0 & 0 & \frac{l_{i}}{E_{i}I_{iz}} \end{array}\right]$$

where *l_i_*, *E_i_S_i_*, *E_i_I_iy_*, *E_i_I_iZ_*, *G_i_J_i_* correspond to the length of the link, tensile stiffness, compression stiffness, *y*-bending stiffness, *z*-bending stiffness and torsional stiffness, respectively.

**Figure 5:**
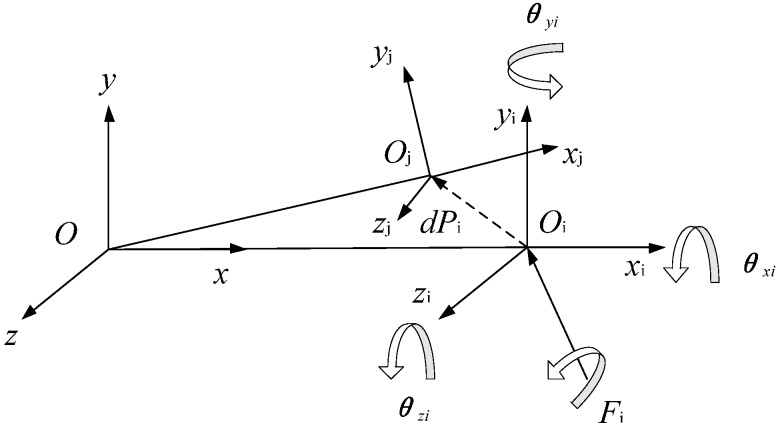
Schematic diagram of deformation of link.

The link is deformed under the external force *Fi* and moment in Figure 5. *Oxyz*, *O_i_x_i_y_i_z_i_*, *O_j_x_j_y_j_z_j_* are the reference coordinate system, link end coordinates before and after deformation, respectively. $l_{i}$ is the length of the link; *F*_i_ link end force, including force and moment. *dP*_i_ is the displacement of end link caused by deformation. *θ_xi_*, *θ_yi_*, *θ_zi_* are the angular displacement caused by deformation. The deformation can be expressed as:

(14) $$\Delta r_{ii}=C_{ii}F_{i}$$

in which $F_{i}={\left(F\;M\right)}^{T}={\left(F_{xi}\;F_{yi}\;F_{zi}\;M_{xi}M_{yi}\;M_{zi}\right)}^{T}$, and $\Delta r_{ii}={\left(dP_{i}\;d\theta_{i}\right)}^{T}={\left(u_{i}\;v_{i}\;w_{i}\;{\theta_{xi}}^{\prime }{\theta_{yi}}^{\prime }\;{\theta_{zi}}^{\prime }\right)}^{T}$. 

The flexibility matrix *C_ii_* for link, expressed in local coordinate system, should be transformed to the global coordinate system to reduce the computation cost as follows:

(15) $$C_{i}=\left\{\begin{array}{cc} R_{oi} & -R_{oi}\left(P_{i4}\times \right)\\ 0 & R_{oi} \end{array}\right\}C_{ii}{\left\{\begin{array}{cc} R_{oi} & -R_{oi}\left(P_{i4}\times \right)\\ 0 & R_{oi} \end{array}\right\}}^{T}$$

where *R_oi_* is rotation matrix of the *i*th member from the local coordinate system to the global coordinate system. *P_i_*_4_ is the link coordinate origin position vector of the *i*th element in local coordinate system. *P_i_*_4_× is the cross product matrix, and can be expressed as:

(16) $$P_{i4}\times =\left[\begin{array}{ccc} 0 & -z & y\\ z & 0 & -x\\ -y & x & 0 \end{array}\right]$$

The flexibility matrix is expressed as:

(17) $$C=\sum_{i=1}^{4}C_{i}$$

There are three generalized forces *F* related to the errors mentioned above, so the required flexibility matrix consists only of the Equation (13) in 3~5 rows and 3~5 columns intersection of elements. The reaction constraint forces and moments in the local coordinate system can be obtained.

(18) $$F_{i}={\left(F\;M\right)}^{T}={\left(0\;0\;F_{zi}\;M_{xi}M_{yi}\;0\right)}^{T}$$

The moving platform is simplified to a revolute pair. Considering the deformation by the forced assembly of the mechanism, the error of the output point [29] is:

(19) $$\begin{array}{ll} dT & ={\left(T_{2}^{0}\right)}^{-1}T_{1}^{0}\Delta_{1}{\Delta_{1}}^{\prime }T_{2}^{1}\Delta_{2}{\Delta_{2}}^{\prime }\\ & \;={\left(T_{1}^{0}T_{2}^{1}\right)}^{-1}T_{1}^{0}\Delta_{1}{\Delta_{1}}^{\prime }T_{2}^{1}\Delta_{2}{\Delta_{2}}^{\prime }={\left(T_{2}^{1}\right)}^{-1}\Delta_{1}{\Delta_{1}}^{\prime }T_{2}^{1}\Delta_{2}{\Delta_{2}}^{\prime } \end{array}$$

where $T_{2}^{0}$ is the ideal position of the output point, and ${\Delta_{i}}^{\prime }=\left[\begin{array}{cccc} 1 & -{\theta_{zi}}^{\prime } & {\theta_{yi}}^{\prime } & u_{i}\\ \;{\theta_{zi}}^{\prime } & 1 & -{\theta_{xi}}^{\prime } & v_{i}\\ -{\theta_{yi}}^{\prime } & {\theta_{xi}}^{\prime } & 1 & w_{i}\\ 0 & 0 & 0 & 1 \end{array}\right]$.

Due to the linkage deformation produced by forced assembly, the energy was stored in the mechanism. This energy is known as elastic deformation energy or strain energy. According to material mechanics, the energy is calculated by the following equation:

(20) $$W=\frac{1}{2}{\left\{\begin{array}{c} z_{0}\\ \alpha_{0}\\ \beta_{0} \end{array}\right\}}^{T}\left\{\begin{array}{ccc} \delta_{11} & \delta_{12} & \delta_{13}\\ \delta_{21} & \delta_{22} & \delta_{23}\\ \delta_{31} & \delta_{32} & \delta_{33} \end{array}\right\}\left\{\begin{array}{c} z_{0}\\ \alpha_{0}\\ \beta_{0} \end{array}\right\}$$

Deformation capacity and flexibility coefficient are varied as the change of position and pose in the working process, resulting in changing the elastic deformation energy of the mechanism.

**Figure 6:**
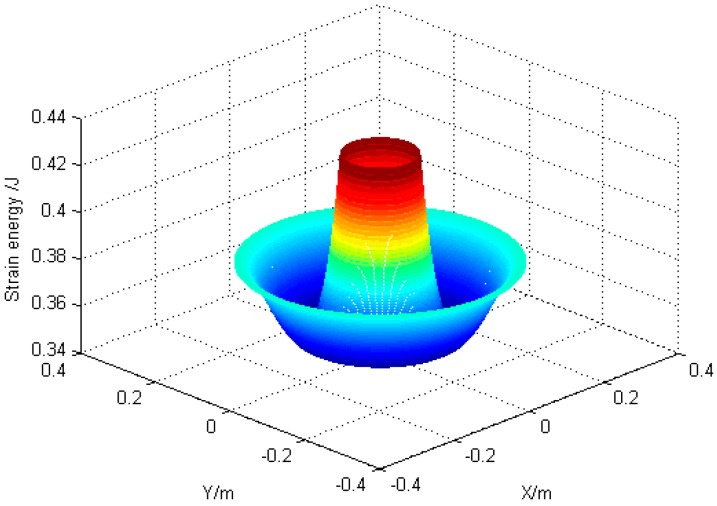
Fluctuation diagram of deformation.

**Figure 7:**
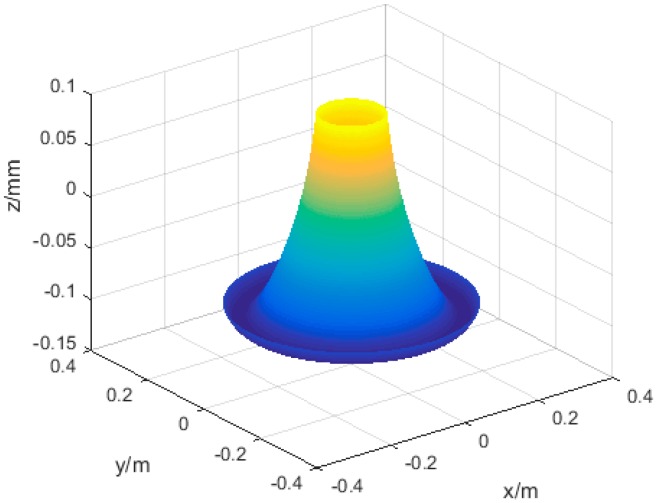
The displacement of the output point along *z*.

According to the design, *r*_1_ = 120 mm, *r*_2_ = 180 mm, *r*_3_ = 60 mm, the torsional stiffness of link is 20,106 N·m^2^, bending stiffness is 25,133 N·m^2^. The axis phase angle and the angle of axis intersection angle is 0.07° and 0.07°, respectively. MATLAB was applied to analyze strain energy of the structure (Figure 6) and displacement along *z* axis (Figure 7). 

## 4. Experimental Study

**Figure 8:**
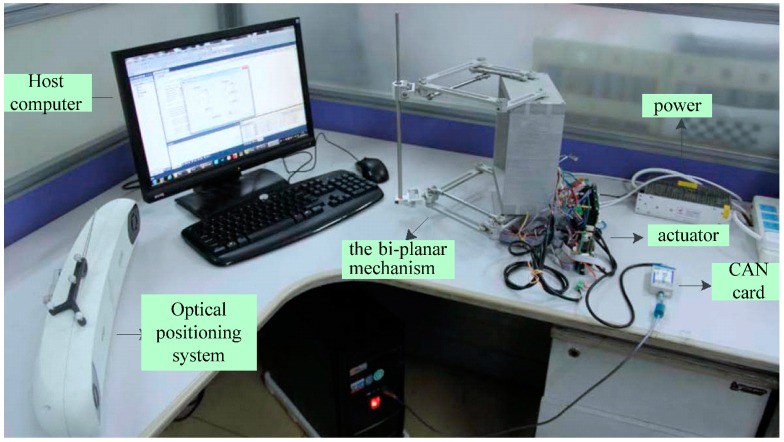
Schematic diagram of the experimental system.

The performance of the bi-planar parallel robot was verified by experiments, including positioning accuracy, orientation accuracy and precision of repetitive positioning. To measure the coordinates of the output points in the up and down platform, NDI Polaris spectra [31] 3D real-time measurement system was used. The prototype of the bi-planar parallel robot is shown in Figure 8, which includes the host computer, CAN card, actuator, the bi-planar mechanism and optical positioning system.

### 4.1. Positioning and Orientation Accuracy Experiment

Firstly, the position coordinates of the lower and up planar 5R mechanism were measured using the NDI Polaris spectra to verify the positioning accuracy of the mechanism. 10 sets of data, theory coordinate value and actual coordinate values are shown in Table 1 and Table 2, respectively.

The total positioning error is *E*, which can be expressed as:

(21) $$E=\sqrt{\Delta x^{2}+\Delta y^{2}+\Delta z^{2}}$$

**Figure 9:**
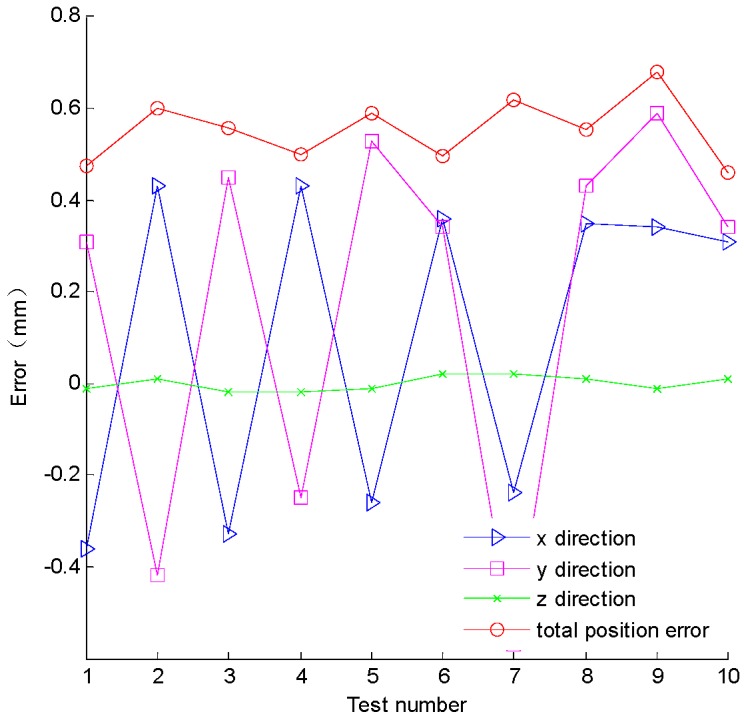
The variation curves of the positioning error.

**Figure 10:**
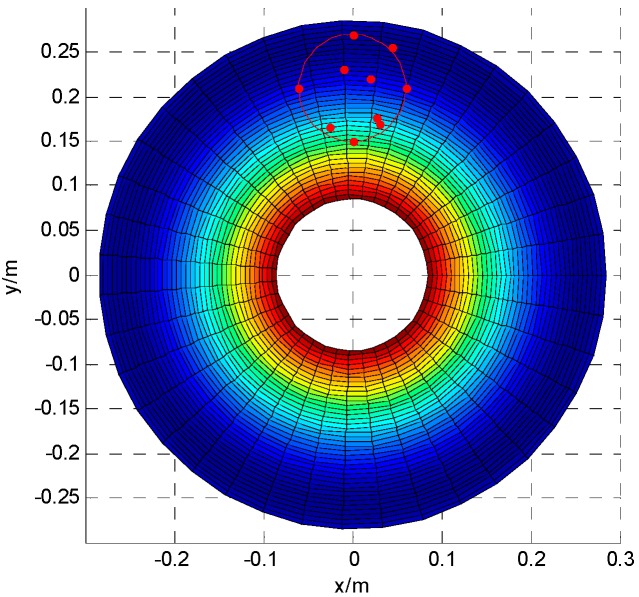
The relationship between the workspace, test point and the positioning error along *z*.

According to the experimental data in Table 1, the position error of each direction and the change of the total positioning error are shown in Figure 9. According to the experiment, the average value of positioning error, *ӯ*, is 0.55 mm. The workspace projection of the bi-planar parallel robot on *xoy* plane is the red circle area in Figure 10. The red points are the experimental points in the working area. The Figure 10 is the projection of the Figure 7 on *xoy* plane. The positioning error along *z* in Figure 9 is consistent with the changes of Figure 7. The total error and error along *x* and *y* directions are basically in agreement with the theoretical error function of Equation (6).

Then, the orientation tracking experiment was carried out by the rigid body (four planar ball trackers) provided in NDI package. The *z*-axis of the rigid-body coordinate is parallel to the *z*-axis of the global coordinate system, and the *x*-axis of the rigid-body coordinate is parallel to the *y*-axis of the global coordinate system. Furthermore the *x* and *z* coordinate values in the rigid-body coordinate system are zero in the global coordinate system. The output point of the up planar 5R mechanism is fixed at (0, 210, 210) mm. The output point of the lower 5R mechanism moves along the theoretical values in Table 1, and then the corresponding orientation errors can be measured. The average value of samples, $\bar{C}$ = 0.15, is used to evaluate the orientation error.

### 4.2. Repetitive Positioning and Repetitive Orientation Precision Experiments

Repetitive positioning accuracy of the bi-planar 5R robot was verified. Here, the definition for repetitive positioning accuracy is the level of inconsistency of the robot position from the same instruction. Assuming the upper and lower planar 5R mechanism of repetitive positioning point is (35, 235), the 20 sets of experimental data, shown in Table 3 and Table 4.

The data is processed according to the normal distribution [32]:

(22) $$\sigma =\sqrt{\frac{1}{N}\sum_{i=1}^{N}{\left(x_{i}-\mu \right)}^{2}}$$

where *x_i_* is the value of each experiment data; *μ* is the true value or accepted reference value of a test property; *N* is the number of experiments.

The calculated results are *u* = 0.6290 and *σ* = 0.0696 mm. *u* + 3*σ* is located in 0.84 mm, while *u* − 3*σ* is located in 0.42 mm. The repetitive positioning accuracy of the mechanism is 0.42 mm in the interval of (0.42, 0.84) mm. The repetitive orientation accuracy of the planar 5R mechanism is 0.17° in the interval of (0.04°, 0.22°). Based on the experimental results, it can be shown that the positioning and orientation accuracy of the bi-planar 5R mechanism are 0.63 mm and 0.15°, respectively. The corresponding repetitive positioning and orientation accuracy are 0.42 mm and 0.18°, respectively. These data meet the technical specification of the micro-part robot for the placement of pedicle screws, namely the target is 0 ± 1 mm in position and 0 ± 1° in orientation.

## 5. Conclusions

The manufacturing and assembly error of the bi-planar parallel mechanism in a pedicle screw robot system have been analyzed. The error transfer function has been formulated to find those geometric errors affecting the pose error, which can predict the motion errors quantitatively. According to the specific accuracy requirement, the amount of the manufacturing and assembling tolerances is obtained.

(1) An error model of the mechanism has been proposed by a complete differential-coefficient theory. In addition, the relations between manufacturing errors, joint angle errors, assembly errors and the position- stance errors of the end effector have been established.
(2) By analyzing the position-stance change of the end effecter, manufacturing errors and joint error have much more effect on the position-stance, so it is necessary to improve the manufacturing and assembly techniques. The trend of the position-stance changing the end effector is nonlinear.
(3) The errors of the mechanism have a great effect on the position-stance of the end effector. Therefore, in the pedicle screw robot system, software and hardware compensations have been applied to correct the position-stance to improve the precision of the parallel mechanism.

## Figures and Tables

**Table 1:** Experimental data of lower planar 5R mechanism position.

Test No.	Theoretical Coordinate Value (*x*, *y*, *z*) (mm)	Measured Coordinate Values (*x*, *y*, *z*) (mm)
1	45.00, 255.00, 0.00	44.64, 255.31, −0.01
2	60.00, 210.00, 0.00	60.43, 209.58, 0.01
3	0.00, 270.00, 0.00	−0.33, 270.45, −0.02
4	−10.00, 230.00, 0.00	−9.57, 229.75, −0.02
5	−60.00, 210.00, 0.00	−60.26, 210.53, −0.01
6	−25.00, 165.00, 0.00	−24.64, 165.34, 0.02
7	0.00, 150.00, 0.00	−0.24, 149.43, 0.02
8	30.00, 170.00, 0.00	30.35, 170.43, 0.01
9	20.00, 220.00, 0.00	20.34, 220.59, −0.01
10	27.00, 176.00, 0.00	27.31, 176.34, 0.01

**Table 2:** Experimental data of the up planar position.

Test No.	Theoretical Coordinate Value (*x*, *y*, *z*) (mm)	Measured Coordinate Values (*x*, *y*, *z*) (mm)
1	45.00, 255.00, 210.00	44.65, 255.24, 209.96
2	60.00, 210.00, 210.00	60.43, 209.74, 210.02
3	0.00, 270.00, 210.00	−0.15, 270.37, 209.95
4	−10.00, 230.00, 210.00	−9.73, 230.41, 209.99
5	−60.00, 210.00, 210.00	−60.27, 210.38, 209.99
6	−25.00, 165.00, 210.00	−25.23, 165.32, 210.01
7	0.00, 150.00, 210.00	−0.22, 149.73, 210.02
8	30.00, 170.00, 210.00	30.32, 170.35, 210.01
9	20.00, 220.00, 210.00	20.16, 219.66, 209.98
10	27.00, 176.00, 210.00	27.23, 175.51, 210.01

**Table 3:** Experiment data of the repetitive positioning accuracy. (Unit: mm)

Test No.	Data	Test No.	Data	Test No.	Data	Test No.	Data
1	0.48	6	0.60	11	0.63	16	0.68
2	0.51	7	0.61	12	0.64	17	0.68
3	0.53	8	0.62	13	0.64	18	0.71
4	0.59	9	0.62	14	0.64	19	0.75
5	0.60	10	0.63	15	0.65	20	0.77

**Table 4:** Experiment data of the repetitive orientation accuracy. (Unit: °)

Test No.	Data	Test No.	Data	Test No.	Data	Test No.	Data
1	0.0808	6	0.1123	11	0.1295	16	0.1507
2	0.0915	7	0.1147	12	0.1370	17	0.1597
3	0.0957	8	0.1185	13	0.1375	18	0.1633
4	0.1023	9	0.1250	14	0.1411	19	0.1672
5	0.1058	10	0.1212	15	0.1425	20	0.1859
